# Hair Trace Element Imbalance in Smokers with HFpEF: A Pilot Study of Micronutrient and Metal Homeostasis

**DOI:** 10.3390/biomedicines14050970

**Published:** 2026-04-23

**Authors:** Beata Krasińska, Tomasz Urbanowicz, Ievgen Spasenenko, Krzysztof J. Filipiak, Krzysztof Bartuś, Zbigniew Krasiński, Andrzej Tykarski, Anetta Hanć

**Affiliations:** 1 Department of Hypertensiology, Angiology, and Internal Medicine, Poznan University of Medical Sciences, 61-848 Poznań, Poland; 2 Cardiac Surgery and Transplantology Department, Poznan University of Medical Sciences, 61-848 Poznań, Poland; 3 The Centre of Postgraduate Medical Education, 01-813 Warsaw, Poland; 4 Department of Cardiovascular Surgery and Transplantology, Jagiellonian University Medical College, 31-202 Kraków, Poland; 5 Department of Vascular, Endovascular Surgery, Angiology and Phlebology, Poznan University of Medical Sciences, 61-848 Poznań, Poland; 6 Department of Trace Analysis, Faculty of Chemistry, Adam Mickiewicz University, 61-614 Poznań, Poland

**Keywords:** HFpEF, trace elements, copper, hair

## Abstract

**Background:** Trace elements function as essential micronutrients involved in oxidative balance, mitochondrial activity, and cardiovascular metabolism. Cigarette smoking represents a significant source of toxic metals and may disrupt systemic trace element homeostasis. Alterations in micronutrient and metal balance may contribute to oxidative stress, endothelial dysfunction, and myocardial remodeling, which are central mechanisms in the pathogenesis of heart failure with preserved ejection fraction (HFpEF). This study aimed to investigate whether smokers with HFpEF exhibit distinct hair trace element profiles compared with smokers without HFpEF. **Methods:** In this prospective pilot study, scalp hair samples were collected from adults undergoing clinical evaluation for suspected cardiovascular disease. Trace element concentrations were determined using inductively coupled plasma mass spectrometry (ICP-MS). Participants were first stratified according to smoking status and subsequently, within the smoker subgroup, according to HFpEF diagnosis based on the Heart Failure Association Pre-test assessment, Echocardiography and natriuretic peptide score (HFA-PEFF) algorithm. Differences in trace element concentrations were analyzed using appropriate statistical tests, with multiple-comparison correction using the Benjamini–Hochberg false discovery rate (FDR). Active smoking was defined as ≥10 cigarettes per day for at least 1 year, and cumulative exposure was quantified in pack-years. **Results:** Fifty-eight participants were included, including 27 active smokers. In unadjusted analyses, several trace elements differed between smokers with HFpEF and those without HFpEF, including vanadium, lithium, aluminum, and copper. However, after FDR correction, only copper remained significantly elevated in smokers with HFpEF (q = 0.004). Hair copper concentrations were markedly higher in the HFpEF group compared with smokers without HFpEF. These differences were observed alongside echocardiographic features consistent with diastolic dysfunction and structural cardiac remodeling. **Conclusions:** In this hypothesis-generating pilot study, smokers with HFpEF demonstrated elevated hair copper concentrations, suggesting disturbances in trace element and micronutrient homeostasis. Altered copper metabolism may reflect oxidative stress-related cardiometabolic remodeling associated with HFpEF. These findings raise the hypothesis that cardiometabolic phenotype, rather than smoking exposure alone, may modulate trace element homeostasis in HFpEF; however, causal relationships cannot be established.

## 1. Introduction

Cigarette smoking remains one of the major modifiable risk factors for cardiovascular disease. Because tobacco smoke contains many different metals and metalloids, it can promote oxidative stress and disturb normal endothelial function. As a result, it may change the distribution of metals in the body and negatively affect mitochondrial activity. Beyond atherosclerosis, chronic exposure to tobacco smoke promotes oxidative stress, systemic inflammation, endothelial dysfunction, and neurohormonal activation—mechanisms implicated in the pathogenesis of heart failure with preserved ejection fraction (HFpEF) [1,2]. Long-term exposure to active smoking or passive inhalation of these elements may disrupt the body’s metal balance, which can influence inflammatory signaling and impair mitochondrial function—both key features in the pathophysiology of HFpEF [3].

HFpEF is characterized by impaired diastolic function, increased myocardial stiffness, and structural remodeling, frequently accompanied by systemic inflammatory activation and microvascular dysfunction [4,5]. Comorbidities such as obesity, hypertension, and metabolic disturbances further contribute to disease progression.

Trace elements represent essential micronutrients involved in mitochondrial respiration, antioxidant defense, and cardiovascular metabolism. Nutritional status and environmental exposure jointly determine systemic trace element homeostasis. Disturbances in micronutrient balance—including copper, zinc, and selenium—have been associated with oxidative stress, endothelial dysfunction, and cardiometabolic disease. Understanding how micronutrient-related trace element dysregulation contributes to heart failure phenotypes may provide new insights into metabolic and inflammatory mechanisms of HFpEF.

Trace elements participate in redox homeostasis and mitochondrial function. Dysregulation of essential and nonessential metals may influence myocardial remodeling and diastolic dysfunction. For example, copper is required for normal antioxidant defense, but when its metabolism becomes dysregulated, it can contribute to redox imbalance and the development of myocardial fibrosis, potentially worsening diastolic function [6]. Experimental studies also indicate that trace-element exposure may act together with oxidative stress to enhance the long-term cardiovascular toxicity associated with smoking.

Hair analysis provides a noninvasive matrix that reflects longer-term systemic exposure [7,8]. We hypothesized that active smokers with HFpEF would demonstrate a distinct trace element profile compared with smokers without HFpEF.

To our knowledge, no previous studies have investigated hair trace element profiles in smokers with HFpEF, despite the potential interaction between environmental exposure, micronutrient imbalance, and cardiometabolic remodeling. The primary endpoint of our analysis was the difference in hair concentrations of selected trace elements between smokers with and without HFpEF.

However, the biological relevance of circulating and tissue trace element alterations in cardiovascular disease remains complex, and whether these changes represent causal mechanisms or epiphenomena of systemic inflammation is still debated.

## 2. Patients and Methods

This prospective pilot study was conducted in 2025 at a tertiary academic center. Patients were consecutively enrolled after referral from cardiology clinics for evaluation of cardiovascular symptoms, including exertional dyspnea, fatigue, and, in some cases, stable angina. A structured patient selection process was applied, and all included participants met predefined inclusion and exclusion criteria.

**Figure 1:**
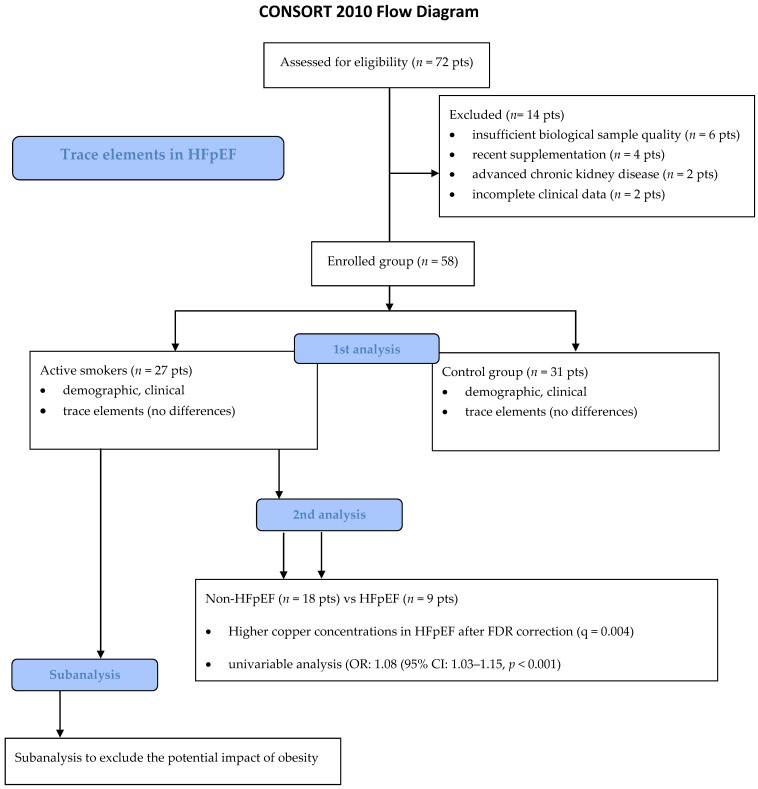
Flow chart.

A total of 72 patients were initially screened for eligibility. Fourteen individuals were excluded due to predefined exclusion criteria (*n* = 6 for insufficient biological sample quality; *n* = 4 for recent supplementation; *n* = 2 for advanced chronic kidney disease, and *n* = 2 for incomplete clinical data). The final study population comprised 58 participants: 27 active smokers and 31 non-smokers. Among active smokers, 9 met criteria for HFpEF and 18 were classified as non-HFpEF, as presented in Figure 1.

Active smoking was defined as the daily consumption of at least 10 cigarettes for a minimum duration of one year. Smoking exposure was quantified in pack-years and included in subsequent analyses as a continuous variable.

### 2.1. Enrolment Criteria

#### 2.1.1. Inclusion Criteria

Adults (≥18 years) electively admitted for evaluation of cardiovascular symptoms, including exertional dyspnea and fatigue, to internal medicine and hypertension clinics were screened.

#### 2.1.2. Exclusion Criteria Included

- Previous cardiovascular interventions;
- Restrictive diet or food allergies;
- Advanced chronic kidney disease (stage 4–5);
- Active malignancy;
- Documented occupational or mental heavy metal exposure exceeding background levels;
- Any supplementation within last 12 months, including copper;
- Insufficient biological samples;
- Quality.

All patients underwent laboratory testing, transthoracic echocardiography, and—when clinically indicated—coronary angiography. Significant coronary artery disease was defined as ≥50% luminal stenosis on visual assessment.

### 2.2. HFpEF Diagnosis

Heart failure with preserved left ventricular ejection fraction (HFpEF) was diagnosed using the Heart Failure Association (HFA)-PEFF diagnostic algorithm [9] by two cardiologists independently, based on:

- Symptoms (exertional dyspnea, fatigue);
- Preserved left ventricular ejection fraction;
- Echocardiographic indices of diastolic dysfunction (E/A ratio, E/e′);
- Increased LAVI
- Reduced GLS;
- Elevated B-type natriuretic peptide (BNP);
- Body mass index (BMI) >30 kg/m^2^, when applicable.

### 2.3. Trace Element Examination

Scalp hair samples were collected according to a standardized protocol [10]. Trace elements examination: hair collection, preparation, and ICP-MS analysis. Hair samples (2–3 cm in length), untreated and free from dyes or chemical processing, were collected from the occipital region close to the scalp. The samples were cleaned by sequential washing in acetone, deionized water, 0.5% Triton X-100, and again deionized water, and then dried. Approximately 150–200 mg of dried hair was used for mineralization in the DigiTube system (SCP Science Ltd., Baie-D’Urfé, QC, Canada). For digestion, 4 mL of 65% nitric acid and 1 mL of 30% hydrogen peroxide were added. The samples were digested in a block system at 150 °C for 4 h. After cooling, the digested material was diluted 100-fold with ultrapure water [11]. The methodology was consistent across all samples to ensure comparability. Element concentrations were measured using an inductively coupled plasma mass spectrometer (ICP-MS, Agilent 7700×, Tokyo, Japan) equipped with a quadrupole mass analyzer and an Octopole Reaction System (ORS). To reduce polyatomic interferences and improve measurement accuracy, the ORS was operated in helium-collision mode. The instrument was tuned each day, and the operating parameters were kept constant: 1550 W RF power, plasma gas flow of 15 L min^−1^, nebulizer gas flow of 0.98 L min^−1^, and auxiliary gas flow of 0.9 L min^−1^. High-purity argon (99.999%) was used as both the carrier and plasma gas. A rhodium internal standard (20 μg L^−1^) was added to all samples and standards to correct for instrumental drift and matrix effects. Mathematical corrections were applied to eliminate any remaining isobaric overlaps. Multi-element calibration curves were prepared in the ranges of 1–500 μg L^−1^ for Ca, Fe, and Mg, and 0.05–100 μg L^−1^ for the other elements, with all correlation coefficients (R) greater than 0.9996. Method performance was verified using the certified reference material for human hair, NCS ZC 8100 2b (CISRI, Beijing, China), processed in the same manner as the moss samples. Limits of detection (LOD) ranged from 0.006 µg/g for Cd to 10 µg/g for Ca. The method showed high precision, with coefficients of variation (CV) of 1.6–3.8%, and good accuracy, with recoveries of 92–108% as previously described [12].

### 2.4. Endpoints

#### 2.4.1. Primary Endpoints

The primary endpoint included trace element hair scalp analysis between the active smokers and patients who reported neither active nor passive nicotine in their medical history.

#### 2.4.2. Secondary Endpoints

The secondary endpoint referred to active smokers who were divided according to HFpEF diagnosis. Hair trace element concentrations were compared between HFpEF patients and a group that did not meet HFpEF criteria.

#### 2.4.3. Tertiary Endpoints

As the HFpEF group had significantly higher BMI values, we performed a further subanalysis to determine whether the secondary endpoint results were related to HFpEF diagnosis or to obesity.

### 2.5. Statistical Analysis

After the normality of the distribution of variables was tested with the Shapiro–Wilk test, the median (IQR) was used to describe the variables that did not follow the normal distribution. We used the *t*-test and the Mann–Whitney test to compare the variables between the groups. Statistical analysis was performed using JASP (Version 0.14.1, University of Amsterdam, Amsterdam, The Netherlands, 2020). Significance was set at *p* < 0.05.

Because multiple trace elements were analyzed simultaneously, the Benjamini–Hochberg false discovery rate (FDR) correction was applied to control for type I error. FDR-adjusted q-values < 0.05 were considered statistically significant.

Primary comparisons included smokers vs. non-smokers regarding demographic, clinical, and hair trace-element concentrations. Thereafter, the smoker group was subdivided by HFpEF vs. non-HFpEF diagnosis. Missing data were handled by pairwise deletion.

To account for potential confounding, an exploratory multivariable regression analysis was performed including age, body mass index, and smoking exposure (pack-years) as covariates, followed by copper concentration with HFpEF status as the dependent variable.

### 2.6. Bioethics Committee Approval

All participants provided written informed consent. The study was approved by the Bioethics Committee of Poznan University of Medical Sciences (protocol no. 411/24; 27 June 2024) and conducted in accordance with the Declaration of Helsinki.

## 3. Results

Fifty-eight patients (32 men [55%]) were included. The median age was 69 years (IQR, 64–76). There were no significant demographic, clinical, or laboratory differences in hair trace element concentrations between smokers and non-smokers, as presented in Table 1.

In hair sample analysis for 21 trace elements, we did not find significant differences between the active smokers and the control group, as presented in Table 2.

In the second step, we investigated the active-smoking population, including 27 participants. Among them, 9 met criteria for HFpEF and 18 were enrolled into the non-HFpEF group, as described in detail in Table 3.

In smokers with HFpEF, scalp hair concentrations of the following elements were significantly higher compared with smokers without HFpEF: Vanadium (*p* = 0.037), Lithium (*p* = 0.007), Aluminum (*p* = 0.018), and Copper (*p* < 0.001), as presented in Table 4.

We performed multiple testing correction using the Benjamini–Hochberg false discovery rate (FDR), revealing the significant difference only in Copper concentration (q = 0.004). The results are presented in Table 4.

The initially observed discrepancy in beryllium concentrations was identified as a unit inconsistency during data verification and has been corrected. After correction, no significant difference in beryllium levels was observed between groups.

**Figure 2:**
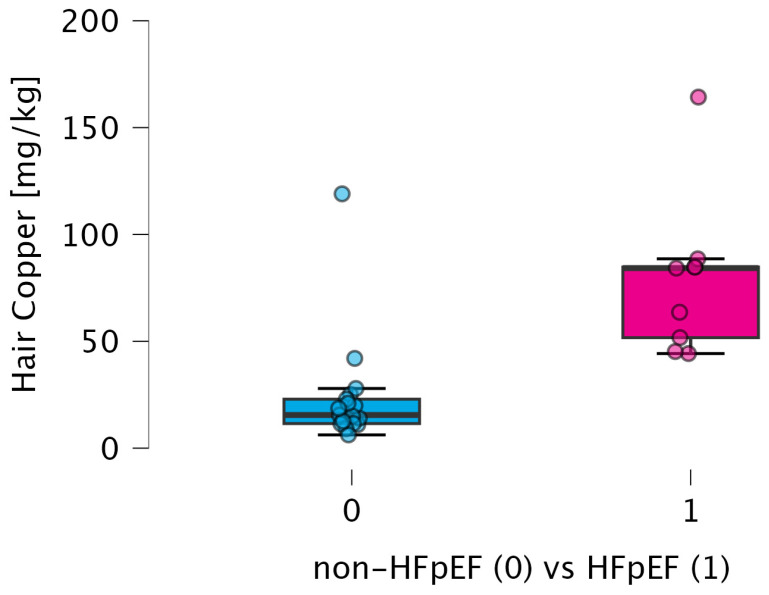
The differences in copper concentrations between HFpEF and non-HFpEF subgroups. Abbreviations: HFpEF—heart failure with preserved ejection fraction, kg—kilogram, mg—milligram.

After adjustment for potential confounders, the association between elevated copper concentration and HFpEF remained directionally consistent, although the study was not powered for definitive multivariable inference. The differences in copper concentration between HFpEF and non-HFpEF subgroups are presented in Figure 2.

### 3.1. Multivariable Regression Analysis

In exploratory uni- and multivariable regression analysis adjusting for age, body mass index, and smoking exposure (pack-years), the association between copper concentration and HFpEF remained directionally consistent. However, the model did not reach statistical significance, likely due to limited sample size as presented in Table 5.

### 3.2. The Possible Role of Obesity

These biochemical differences were observed in parallel with echocardiographic evidence of structural and functional alterations, meeting the HFpEF criteria combined with clinical characteristics. To assess whether the observed differences were driven by obesity rather than HFpEF, we performed an additional subgroup analysis stratified by BMI. The active smoker group was subdivided into patients with a BMI over 30 kg/m^2^ and the non-obese population. Hair concentrations of 21 trace elements were investigated, and no significant differences in scalp trace element concentrations were observed (Table 6).

## 4. Discussion

In this prospective pilot analysis of active smokers, we observed differences in scalp hair trace element concentrations between individuals with HFpEF and smokers without evidence of diastolic heart failure. In unadjusted analyses, several trace elements differed between smokers with and without HFpEF, including vanadium, lithium, aluminum, and copper. However, after adjustment for multiple comparisons using the Benjamini–Hochberg FDR procedure, only copper remained statistically significant. The selective elevation of copper, in contrast to other trace elements, may reflect its unique role in oxidative stress pathways and extracellular matrix remodeling [13,14]. Copper is actively incorporated into hair through binding to keratin-associated proteins and may accumulate under conditions of altered systemic distribution, particularly in states of chronic inflammation [15,16].

These findings suggest that alterations in trace element homeostasis may accompany the structural and functional myocardial changes characteristic of HFpEF in smokers. The analysis was based on hair samples, which reflect long-term exposure rather than short-term fluctuations in plasma. The primary endpoint was not significant; therefore, findings should be interpreted cautiously as secondary/exploratory.

Tobacco smoking remains a challenging problem with a significant impact on human morbidity and mortality [17,18]. Its impact on cardiovascular diseases, particularly premature atherosclerosis, is a major concern [19]. Cigarette smoke is a complex mixture containing numerous metals and metalloids capable of inducing oxidative stress and endothelial dysfunction [20]. Chronic exposure to active smoking or passive inhalation of these elements may alter systemic metal distribution, leading to altered inflammatory signaling and mitochondrial function [21]. Previous studies [22,23] highlighted dysregulation of several trace element concentrations, including copper, cadmium, and selenium, reflecting both environmental exposure and altered metabolic handling of metals, resulting in cardiovascular pathology. Such disturbances may contribute to the mechanisms recognized as central components of HFpEF pathophysiology. The lack of differences in trace element concentrations between smokers and non-smokers contrasts with previous reports [24,25]. This discrepancy may reflect differences in population characteristics, analytical methods, or the relatively small sample size of the present study. Importantly, the absence of differences between smokers and non-smokers suggests that smoking alone does not explain the observed copper elevation. Instead, HFpEF-related systemic changes, including inflammation and metabolic dysregulation, may play a dominant role [12,26,27].

In our study, which focused on the active-smoker population, copper showed the most consistent association with the HFpEF group. Several elements differed in our unadjusted comparisons; however, after FDR correction, only copper remained significantly elevated. The marked increase in hair copper levels observed in HFpEF patients (84.26 mg/kg vs. 15.00 mg/kg, *p* < 0.001; Table 4) becomes even more relevant when considered alongside the echocardiographic signs of structural and functional impairment shown in Table 3. In particular, the HFpEF group demonstrated lower global longitudinal strain (GLS) (14% vs. 19%, *p* < 0.001) and higher E/E’ ratios (11 vs. 8, *p* < 0.001), indicating reduced myocardial deformation and elevated left ventricular filling pressures. These findings should be interpreted within the framework of systemic inflammatory–resolution coupling rather than direct causality.

Copper is one of the essential trace elements that play pivotal roles in mitochondrial respiration and antioxidant defense [28]. Copper is a key micronutrient involved in mitochondrial respiration, oxidative phosphorylation, and antioxidant defense through enzymes such as superoxide dismutase and cytochrome c oxidase. Dysregulated copper homeostasis has been linked to cardiovascular remodeling, myocardial fibrosis, and metabolic dysfunction. Elevated copper levels may reflect altered systemic regulation of micronutrients under conditions of chronic inflammation and oxidative stress. Its dysregulated metabolism may contribute to myocardial remodeling and fibrosis by promoting oxidative stress and redox imbalance [29]. Copper plays an essential role as a cofactor for lysyl oxidase (LOX), the enzyme that enables collagen cross-linking within the extracellular matrix. When copper balance is disturbed, fibroblast activity may increase, leading to greater collagen deposition and myocardial stiffening. The incorporation of copper into hair shafts occurs during keratinization and reflects circulating bioavailable copper levels over time [30,31]. Increased deposition may therefore represent systemic redistribution rather than localized scalp-specific processes.

This mechanism aligns with our findings, where patients showed reduced septal and lateral E’ velocities, as presented in Table 3. Excessive intracellular copper can enhance reactive oxygen species generation and impair endothelial function [32], processes that may accelerate myocardial diastolic dysfunction. Elevated circulating copper concentrations have been associated with increased cardiovascular risk and adverse cardiac remodeling [33]. The markedly higher copper concentrations observed in our analysis among smokers with HFpEF may represent a marker of chronic oxidative stress or altered trace element homeostasis rather than direct cardiotoxicity. The absence of significant differences between smokers and non-smokers in the primary analysis suggests that HFpEF status, rather than smoking exposure alone, may be associated with alterations in trace element distribution. Moreover, the additional analysis stratified by obesity did not reveal differences in trace element concentrations, suggesting that the observed copper signal was unlikely to be explained solely by body mass index. As shown in Table 6, none of the 21 analyzed elements—including copper (*p* = 0.777)—differed significantly between obese and non-obese smokers. This supports the idea that the copper imbalance observed in our study is more closely related to heart failure and its accompanying systemic inflammatory response, rather than to metabolic alterations associated with excess body weight. Nonetheless, these findings should be interpreted cautiously, given the limited sample size. Moreover, dietary intake, except for dietary supplementation (including copper supplements), was not systematically assessed, which represents a limitation of our analysis.

Cigarette smoke contains numerous metals and metalloids capable of promoting oxidative stress [34]. Chronic exposure may alter systemic trace element distribution and redox balance. HFpEF is increasingly recognized as a syndrome characterized by systemic inflammation and coronary microvascular dysfunction, conditions that may influence metal handling, tissue redistribution, and excretion. This suggests that the homeostatic disturbances of these trace elements may be closely linked to the degree of structural remodeling and diastolic dysfunction observed in this population. Experimental data [35] further suggest a synergistic interaction between trace-element exposure and oxidative stress in mediating long-term cardiovascular toxicity associated with smoking. These findings should be interpreted as exploratory and hypothesis-generating, and larger studies integrating circulating and tissue trace element profiling are required to clarify the role of metal homeostasis in HFpEF.

From a nutritional perspective, trace element profiling may help identify micronutrient imbalance associated with cardiometabolic disease. If confirmed in larger studies, monitoring trace element status could provide insights into nutritional and environmental determinants of HFpEF pathophysiology.

### Study Limitation

This study has several important limitations. First, the relatively small sample size and single-center design limit generalizability. Second, the absence of a healthy control group precludes conclusions regarding disease-specific alterations. Third, the observational design does not allow causal inference. Fourth, although exploratory multivariable adjustment was performed, residual confounding cannot be excluded. Fifth, dietary intake, renal function, and environmental exposure were not comprehensively assessed. Finally, hair analysis reflects long-term exposure but may not directly correspond to myocardial or intracellular concentrations.

Additionally, no formal sample size calculation was performed due to the pilot nature of the study, which further limits statistical power and the ability to detect smaller effect sizes.

A data inconsistency related to beryllium concentrations was identified and corrected during revision, highlighting the importance of careful interpretation of trace-level measurements.

## 5. Conclusions

In this exploratory pilot study, elevated hair copper concentrations were observed in smokers with HFpEF. These findings raise the hypothesis that trace element imbalance may reflect systemic cardiometabolic remodeling rather than smoking exposure alone. Further studies are required to determine whether copper represents a biomarker or a mechanistic contributor to HFpEF.

## Figures and Tables

**Table 1:** Demographic and clinical characteristics followed by laboratory and echocardiographic results in Active smokers and the control group.

Parameters	Active Smokers *n* = 27 (Median (Q1–Q3))	Control Group *n* = 31 (Median (Q1–Q3))	*p*-Value
Demographic:			
Age (years) (median (Q1–Q3))	69 (61–74)	70 (66–76)	0.57
Sex (males (%))	12 (44)	20 (65)	0.13
BMI (kg/m^2^) (median (Q1–Q3))	30.2 (28.6–32.1)	29.3 (26.7–32.4)	0.69
BMI > 30 kg/m^2^ (*n* (%))	14 (52)	11 (35)	0.22
NYHA status:			
1/II (*n* (%))	6 (22)	8 (26)	1.000
II (*n* (%))	11 (41)	18 (58)	0.292
II/III (*n* (%))	7 (26)	4 (13)	0.315
III (*n* (%))	3 (11)	1 (3)	0.329
Co-morbidities			
Arterial hypertension (*n* (%))	26 (96)	26 (84)	0.22
Hypercholesterolemia (*n* (%))	26 (96)	30 (97)	1.00
Diabetes mellitus (*n* (%))	7 (26)	7 (23)	0.17
PAD (*n* (%))	3 (11)	7 (23)	0.26
COPD (*n* (%))	4 (15)	4 (13)	0.85
Nicotine:			
(packs/year) (median (Q1–Q3))	30 (21–43)	0	<0.001
Laboratory testing:			
WBC (10 × 9/L) (median (Q1–Q3))	6.81 (6.01–7.95)	7.36 (5.64–8.50)	0.53
Hb (mmol/L) (median (Q1–Q3))	8.80 (8.35–9.45)	8.70 (8.05–9.10)	0.25
Plt (10 × 9/L) (median (Q1–Q3))	231 (194–279)	234 (185–275)	0.99
			
Creatinine (umol/L) (median (Q1–Q3))	85 (73–96)	81 (70–94)	0.72
			
ALT (IU/L) (median (Q1–Q3))	30 (23–39)	24 (20–32)	0.69
			
Total cholesterol (mmol/L) (median (Q1–Q3))	3.93 (3.18–5.26)	4.00 (3.53–4.36)	0.53
			
BNP (pg/mL) (median (Q1–Q3))	152 (128–178)	111 (86–218)	0.12
			
Hb1Ac (%) (median (Q1–Q3))	5.8 (5.7–6.0)	5.7 (5.6–5.9)	0.40
			
Ck-MB (ng/mL) (median (Q1–Q3))	1.71 (1.03–2.80)	1.74 (1.18–2.87)	0.74
Echocardiography:			
LVED (mm) (median (Q1–Q3))	49 (43–51)	46 (42–50)	0.81
LVEF (%) (median (Q1–Q3))	56 (52–59)	54 (46–59)	0.79

Abbreviations: %—percentage, ALT—alanine aminotransferase, BMI—body mass index, BNP—B-type natriuretic peptide, Ck-MB—creatine kinase myocardial band, COPD—chronic obstructive pulmonary disease, Hb—hemoglobin, HbA1c—glycated hemoglobin, IU/L—international units per liter, L—liter, mmol/L—millimoles per liter, LVED—left ventricular end diastolic diameter, LVEF—left ventricular ejection fraction, *n*—number of patients, ng/mL—nanograms per milliliter, NYHA—New York Heart Association functional classification, PAD—peripheral artery disease, pg/mL—picograms per milliliter, Plt—platelet count, Q1–Q3—first and third quartiles (interquartile range, IQR), WBC—white blood cell count.

**Table 2:** The hair trace element concentrations in active smokers and the control group.

No	Trace Element Concentration [mg/kg]	Active Smokers *n* = 27 (Median (Q1–Q3))	Control Group *n* = 31 (Median (Q1–Q3))	*p*-Value
1	Mn	0.16 (0.12–0.43)	0.19 (0.11–0.37)	0.581
2	Co	0.017 (0.011–0.024)	0.012 (0.010–0.028)	0.622
3	Fe	7.17 (5.06–16.97)	7.59 (5.43–11.71)	0.846
4	Be	0.001 (0.000–0.004)	0.001 (0.000–0.002)	0.219
5	Cd	0.010 (0.007–0.023)	0.014 (0.009–0.024)	0.822
6	Zn	110.7 (54.7–204.4)	142.4 (93.3–158.8)	0.911
7	V	0.034 (0.025–0.052)	0.043 (0.029–0.062)	0.830
8	Cr	0.90 (0.65–1.21)	0.99 (0.80–1.30)	0.138
9	Na	135 (95–159)	147 (113–179)	0.276
10	Pb	0.193 (1.00–0.326)	0.257 (0.142–0.563)	0.713
11	Ca	291.3 (73.8–1147.9)	308.0 (89.5–972.5)	0.317
12	Mo	0.128 (0.104–0.176)	0.153 (0.120–0.187)	0.173
13	Li	0.016 (0.006–0.029)	0.017 (0.008–0.020)	0.059
14	Sr	0.615 (0.150–3.101)	0.419 (0.220–2.723)	0.878
15	Mg	16.4 (7.4–48.0)	14.9 (7.1–37.5)	0.521
16	Al	5.32 (2.98–9.25)	4.23 (2.91–6.49)	0.514
17	Se	0.362 (0.196–0.750)	0.423 (0.339–0.710)	0.420
18	As	0.027 (0.020–0.053)	0.034 (0.024–0.068)	0.959
19	Cu	15.51 (9.99–27.19)	15.43 (10.44–24.81)	0.661
20	U	0.003 (0.002–0.009)	0.003 (0.002–0.009)	0.525
21	Ni	0.86 (0.74–1.02)	1.00 (0.80–1.44)	0.128

Abbreviations: Al—Aluminum, As—Arsenic, Be—Beryllium, Ca—Calcium, Cd—Cadmium, Co—Cobalt, Cr—Chromium, Cu—Copper, Fe—Iron, Li—Lithium, Mg—Magnesium, Mn—Manganese, Mo—Molybdenum, Na—Sodium, Ni—Nickel, Pb—Lead, Se—Selenium, Sr—Strontium, U—Uranium, V—Vanadium, Zn—Zinc.

**Table 3:** Demographic and clinical characteristics. Laboratory and echocardiographic results.

Parameters	Non-HFpEF *n* = 18	HFpEF *n* = 9	*p*
Demographic:			
Age (years) (median (Q1–Q3))	72 (69–75)	72 (67–76)	1.000
Sex (males (%))	14 (78)	9 (100)	0.565
BMI (kg/m^2^) (median (Q1–Q3))	30.3 (26.4–31.9)	30.4 (26.4–32.0)	0.797
BMI > 30 kg/m^2^ (*n* (%))	10 (56)	5 (56)	1.000
NYHA status:			
I/II (*n* (%))	6 (33)	0 (0)	0.063
II (*n* (%))	11 (61)	0 (0)	0.002
II/III (*n* (%))	1 (6)	6 (67)	0.003
III (*n* (%))	0 (0)	3 (33)	0.034
Co-morbidities			
Arterial hypertension (*n* (%))	17 (94)	9 (100)	1.000
Hypercholesterolemia (*n* (%))	17 (94)	9 (100)	1.000
Diabetes mellitus (*n* (%))	4 (22)	1 (11)	0.516
PAD (*n* (%))	1 (6)	3 (33)	0.066
COPD (*n* (%))	3 (17)	1 (11)	0.738
Nicotine:			
(packs/year) (median (Q1–Q3))	28 (16–32)	40 (30–45)	0.103
Laboratory testing:			
WBC (10 × 9/L) (median (Q1–Q3))	7.4 (6.2–10.0)	7.4 (6.5–7.7)	0.425
Hb (mmol/L) (median (Q1–Q3))	9.0 (8.1–9.4)	8.0 (8.0–8.3)	0.083
Plt (10 × 9/L) (median (Q1–Q3))	229 (183–282)	244 (233–249)	0.292
			
Creatinine (µmol/L) (median (Q1–Q3))	90 (76–98)	73 (67–79)	0.084
			
ALT (IU/L) (median (Q1–Q3))	26 (22–37)	21 (15–29)	0.122
			
Total cholesterol (mmol/L) (median (Q1–Q3))	3.9 (3.1–4.6)	4.2 (3.7–4.3)	0.857
			
BNP (pg/mL) (median (Q1–Q3))	112 (78–178)	331 (256–498)	<0.001
			
Hb1Ac (%) (median (Q1–Q3))	5.8 (5.5–6.3)	5.6 (5.6–5.8)	0.409
			
Ck-MB (ng/mL) (median (Q1–Q3))	2.1 (1.4–3.0)	1.7 (0.7–4.2)	1.000
Echocardiography:			
Septal E’ (median (Q1–Q3))	0.08 (0.06–0.09)	0.06 (0.05–0.07)	<0.001
Lateral E’ (median (Q1–Q3))	0.08 (0.06–0.09)	0.06 (0.05–0.07)	0.032
E/E’ (median (Q1–Q3))	8 (7–14)	11 (8–11)	<0.001
GLS (median (Q1–Q3))	19 (17–21)	14 (12–16)	<0.001
LAVI (median (Q1–Q3))	28 (24–35)	37 (34–40)	<0.001
LMV index (median (Q1–Q3))	88 (81–107)	105 (98–116)	0.014
RWT (median (Q1–Q3))	0.40 (0.38–0.52)	0.43 (0.37–0.54)	0.510
LVEF (%) (median (Q1–Q3))	54 (52–60)	55 (52–58)	0.612

Abbreviations: %—percentage, ALT—alanine aminotransferase, BMI—body mass index, BNP—B-type natriuretic peptide, Ck-MB—creatine kinase myocardial band, COPD—chronic obstructive pulmonary disease, E/E′ ratio—ratio of early transmitral inflow velocity (E wave) to early diastolic mitral annular velocity (E′); estimate of left ventricular filling pressure, E′ (E prime)—early diastolic mitral annular velocity measured by tissue Doppler imaging, GLS—global longitudinal strain, Hb—hemoglobin, HbA1c—glycated hemoglobin, IU/L—international units per liter, L—liter, Lateral E′—early diastolic velocity at the lateral mitral annulus, LAVI—left atrial volume index, LVEF—left ventricular ejection fraction, mmol/L—millimoles per liter, µmol/L—micromoles per liter, *n*—number of patients, ng/mL—nanograms per milliliter, NYHA—New York Heart Association functional classification, PAD—peripheral artery disease, pg/mL—picograms per milliliter, Plt—platelet count, Q1–Q3—first and third quartiles (interquartile range, IQR), RWT—relative wall thickness, Septal E′—early diastolic velocity at the septal mitral annulus, WBC—white blood cell count.

**Table 4:** Hair scalp trace elements analysis in active smokers with HFpEF and nonHFpEF subgroups.

No	Trace Element Concentration [mg/kg]	Non-HFpEF Group *n* = 18 (Median (Q1–Q3))	HFpEF Group *n* = 9 (Median (Q1–Q3))	*p*-Value	FDR (q)
1	Mn	0.20 (0.19–0.32)	0.20 (0.15–1.59)	0.914	0.635
2	Co	0.020 (0.010–0.023)	0.027 (0.010–0.062)	0.191	0.959
3	Fe	8.00 (6.00–11.40)	9.20 (7.73–12.19)	0.518	0.297
4	Be	0.001 (0.000–0.003)	0.001 (0.000–0.002)	0.121	0.319
5	Cd	0.013 (0.010–0.020)	0.020 (0.010–0.068)	0.298	0.457
6	Zn	128 (111–143)	144 (118–535)	0.131	0.172
7	V	0.040 (0.030–0.042)	0.070 (0.060–0.098)	0.037	0.194
8	Cr	0.97 (0.70–1.00)	1.00 (0.84–1.03)	0.342	0.791
9	Na	132 (111–147)	143 (121–153)	0.306	0.428
10	Pb	0.300 (0.190–0.700)	0.460 (0.350–1.440)	0.256	0.457
11	Ca	511 (227–1340)	698 (376–6181)	0.145	0.102
12	Mo	0.142 (0.100–0.170)	0.150 (0.140–0.165)	0.383	0.635
13	Li	0.014 (0.010–0.020)	0.024 (0.020–0.040)	0.007	0.073
14	Sr	1.70 (0.54–2.71)	3.70 (0.40–11.00)	0.235	0.432
15	Mg	22.0 (14.2–57.8)	41.2 (14.9–198.5)	0.281	0.816
16	Al	3.8 (3.3–6.3)	10.0 (4.5–11.1)	0.018	0.128
17	Se	0.410 (0.300–0.500)	0.510 (0.400–0.780)	0.152	0.297
18	As	0.030 (0.024–0.051)	0.030 (0.024–0.070)	0.786	0.636
19	Cu	15.00 (11.50–19.88)	84.26 (51.70–94.76)	<0.001	0.004
20	U	0.004 (0.002–0.010)	0.010 (0.003–0.040)	0.129	0.319
21	Ni	0.91 (0.80–1.00)	1.08 (0.99–1.20)	0.088	0.319

Abbreviations: Al—Aluminum, As—Arsenic, Be—Beryllium, Ca—Calcium, Cd—Cadmium, Co—Cobalt, Cr—Chromium, Cu—Copper, Fe—Iron, Li—Lithium, Mg—Magnesium, Mn—Manganese, Mo—Molybdenum, Na—Sodium, Ni—Nickel, Pb—Lead, Se—Selenium, Sr—Strontium, U—Uranium, V—Vanadium, Zn—Zinc.

**Table 5:** Univariate and multivariable logistic regression analysis for HFpEF. Multivariable model adjusted for age, body mass index, and smoking exposure (pack-years). Due to the limited sample size, the model was considered exploratory.

Variable	Univariable			Multivariable		
	OR	95% CI	*p*-Value	OR	95% CI	*p*-Value
Age	1.02	0.98–1.07	0.31	1.01	0.96–1.06	0.58
BMI	1.05	0.92–1.19	0.44	1.03	0.89–1.18	0.67
Pack-years	1.01	0.99–1.03	0.28	1.00	0.98–1.03	0.61
Copper	1.08	1.03–1.15	<0.001	1.04	0.98–1.12	0.12

Abbreviations: BMI—body mass index, CI—confidence interval, OR—odds ratio.

**Table 6:** Hair scalp trace elements analysis in obese and non-obese subgroups.

No	Trace Element Concentration [mg/kg]	Non-Obese Group *n* = 12 (Median (Q1–Q3))	Obese Group *n* = 15 (Median (Q1–Q3))	*p*-Value
1	Mn	0.30 (0.20–0.44)	0.20 (0.16–0.29)	0.288
2	Co	0.023 (0.020–0.030)	0.013 (0.010–0.035)	0.177
3	Fe	8.00 (6.00–11.40)	9.20 (7.73–12.19)	0.303
4	Be	0.001 (0.000–0.003)	0.001 (0.000–0.002)	0.809
5	Cd	0.010 (0.010–0.023)	0.015 (0.010–0.030)	0.284
6	Zn	137 (99–149)	125 (115–189)	0.662
7	V	0.040 (0.030–0.042)	0.070 (0.060–0.098)	0.437
8	Cr	1.02 (0.73–1.09)	0.99 (0.82–1.00)	0.392
9	Na	141 (112–156)	127 (120–146)	0.738
10	Pb	0.390 (0.240–0.725)	0.375 (0.163–1.255)	0.877
11	Ca	511 (227–1340)	698 (376–6181)	0.938
12	Mo	0.158 (0.116–0.200)	0.140 (0.103–0.158)	0.298
13	**Li**	0.020 (0.010–0.025)	0.016 (0.010–0.020)	0.390
14	Sr	2.05 (0.40–2.89)	1.90 (0.85–3.93)	0.235
15	Mg	22.8 (9.9–62.3)	31.5 (16.4–62.6)	0.425
16	Al	6.7 (3.6–11.1)	4.4 (3.5–5.8)	0.269
17	Se	0.410 (0.300–0.500)	0.510 (0.400–0.780)	0.680
18	As	0.045 (0.030–0.070)	0.030 (0.024–0.045)	0.146
19	Cu	20.44 (12.33–54.27)	21.87 (14.25–60.63)	0.777
20	U	0.008 (0.004–0.040)	0.004 (0.003–0.009)	0.245
21	Ni	0.91 (0.80–1.00)	1.08 (0.99–1.20)	0.756

Abbreviations: Al—Aluminum, As—Arsenic, Be—Beryllium, Ca—Calcium, Cd—Cadmium, Co—Cobalt, Cr—Chromium, Cu—Copper, Fe—Iron, Li—Lithium, Mg—Magnesium, Mn—Manganese, Mo—Molybdenum, Na—Sodium, Ni—Nickel, Pb—Lead, Se—Selenium, Sr—Strontium, U—Uranium, V—Vanadium, Zn—Zinc.

## Data Availability

Data supporting the reported results can be obtained upon reasonable request by contacting the corresponding authors, due to privacy or ethical restrictions.
